# Essay on Spontaneous Human Combustion

**Published:** 1828-06

**Authors:** M. Marc


Art. III.—*On the Spontaneous Combustion of the Human Body.* By M. Marc. *Translated from the Dictionaire des Sciences Medicales, for the Western Journal.* By the Editor.

When we observe the considerable quantity of wood, or other inflammable materials, which is required for the combustion of a corpse, and how slowly that operation proceeds, it seems almost incredible, that it should be effected in a living body, in a few minutes, at the ordinary temperature, and without the known contact of fire. Nothing, however, is more certain than this event; which deserves to be contemplated with the greatest care by physicians, and, especially, by those who devote themselves to the study of legal medicine. In fact, this subject is involved in almost every inquiry into the causes of doubtful death; and it is important to know by what signs, it may be proved to have taken place; that we may not attribute to others, that which occurred independently of all human agency, and was entirely the effect of physical causes. The case narrated by the celebrated Lecat, in a posthumous memoir on spontaneous human combustion, (*Journal of Med. Chir. Phar. &c. for Jan. and Feb.* 1793) is well adapted to the support of this proposition. In 1725, a woman named Millet, an inhabitant of Reims, perished from spontaneous combustion. Her remains were found in the kitchen, about a foot and a half from the fire place; and consisted, only, of a part of the head, of the inferior extremities, and some of the dorsal vertebræ. As there was in the family a young and beautiful female servant, the husband was charged with having destroyed his wife, on her account. He was subjected to a rigorous criminal prosecution and found guilty; but, appealing to a higher and more enlightened tribunal, the case was pronounced to be one of spontaneous combustion; and thus was an innocent man preserved from infamy and the scaffold.

But we need not depend on the records of a period, characterised by the credulity which is inseparable from an imperfect knowledge of the physical sciences, for proofs of the reality of the phenomenon under consideration; as subsequent and better informed ages, have furnished examples so numerous and well authenticated, that we are no longer at liberty to cherish a doubt. *Some of these cases are given, in another volume of this Dictionary, under the head of* Cas Rares.

Dupont, Adolphi and Lecat seem to be the first who made publications on this subject; but it was reserved for M. Lair, at a later period, to illustrate it by a most comprehensive assemblage of facts, and ingenious reasonings. Nevertheless, the principal conclusions of this estimable author have been combatted by professor Kopp, of Hanau, in an excellent monograph which he has published on this subject. It is chiefly from the writings of these Savans, that I shall extract what I am about to say.

Messrs. Lair and Kopp have collected the best authenticated and most conclusive examples of human combustion, and to their works will I refer for these details. There is a case, however, which M. Lair has omitted, that deserves to be related here; seeing that the patient survived the catastrophy for some time, and was able to state the circumstances which preceded and followed it. This case, which M. Joseph Battaglia, a surgeon of Ponte-Bosio, published in one of the Journals of Florence, from whence Dr. Fouquet extracted it into the *Bibliotheque Salutaire* of Paris, 1787, is so much the more worthy of introduction here, as it throws a great deal of light upon the occasional cause of the phenomenon which we are considering.

"Don G. Maria Bertholi, a priest, of Mount Valere, in the district of Livizzano, made a visit, says M. Battaglia, to the fair of Filetto, where he had business. After having devoted a day's journey to the neighboring country, executing some commissions, he pushed forward in the evening for Fenile, and reached the house of his brother-in-law who resided there. On entering it, he requested to be shown into the apartment where he was to lodge. For some reason he placed a handkerchief between his shoulders and his shirt, and every body having withdrawn, he proceeded to say his breviary. But a few minutes had elapsed, when the family heard an extraordinary noise in the room, where they had left him alone. Perceiving the cries of the priest, they repaired instantly to the apartment; and, on entering it, found him extended on the paved floor, and enveloped in a thin flame, which receded as they approached him, and at length entirely vanished. They placed him, as soon as possible, upon his bed, and administered to him every kind of aid that was at hand. On the following day I was called in, and, having examined him with care, I found that the skin of the upper part of his right arm was almost entirely detached from the flesh, and hanging loose. In the space comprised between the shoulder and the thigh, the integuments were also as much injured as those of the arm. I proceeded to remove the fragments of skin, and perceiving an incipient gangrene, upon that part of the right hand which had suffered most, I immediately scarified it; but, in spite of this precaution, I found it the next day, as I had feared, in a state of complete mortification.

"At my third visit, all the other injured parts were equally mortified. The patient complained of intense thirst, and was agitated with horrible convulsions. He discharged, by stool, a quantity of bilious and putrid matter, and was besides harassed with a continual vomiting; accompanied by a great deal of fever and delirium.

"At the end of the fourth day, after two hours of deep stupor, he expired. At my last visit, during which he fell into the lethargy of which I have spoken, I observed with astonishment, that the putrefaction had already made such progress, that the body of the patient exhaled an insupportable stench: worms had been generated in his skin, and were to be seen crawling even on the outside of the bed; and his finger nails of the left hand had pealed off. In this deplorable situation, I thought that nothing more could be attempted with advantage to the patient, and he was left to his fate.

"Having taken care to learn from the patient himself all that passed at the time of the attack, he informed me that when it occurred, he felt as if he had received a blow from a club on his right arm; and, at the same moment, he observed a blue flame issuing from his shirt, which, in an instant, was reduced to ashes, without the fire extending to the wristbands. The handkerchief which, on his arrival, he had applied over his shoulders, between his shirt and skin, remained entirely uninjured; as did, likewise, his drawers; but his cap was wholly consumed, without a single hair of his head being scorched.

"That the elementary fire dispersed in the air, had burnt his skin, reduced his shirt to ashes, and consumed his cap, without touching his hair, in the slightest degree, are facts, which I present to the public as well ascertained, and perfectly certain: besides, all the symptoms of the patient were those of a severe burn. The night was calm, and the air extremely pure. The persons who entered the room, at the time of the accident, did not perceive any bituminous or empyreumatic odour; nor did they observe any smoke. The lamp, previously full of oil, was dry, and the wick in a state of incineration.

In attempting to account for human combustion, we should never lose sight of the fact that it takes place in the living body; and, consequently, that we must reject every explanation, that is exclusively conformable to the chemical and physical laws, which govern bodies deprived of life. It is on this principle, that the reasonings of M. Kopp, must prevail over those of M. Lair.

From observations continued down to the present day, it appears that nearly all the victims of human combustion have been addicted to the excessive use of spirituous liquors. M. Lair has concluded, therefore, that the different parts of their bodies, having suffered an alcoholic impregnation, had contracted a degree of combustibility, which rendered them liable to be burnt up. This opinion, adopted by many physicians, and especially by Beldoes, seemed, at first, to derive support from the dissection of persons, dead from drunkenness, from whose bodies there issued a spirituous odour. M. Lair has added, also, that the flame observed in these combustions, absolutely resembled that of burning alcohol; that the individuals to whom these accidents have happened, were generally very fat or very lean; and that in the former case, the fat supplied fuel for the flame, while, in the latter, a defect of moisture favoured the combustion.

But on the principles of sound physiology, can we admit the assimilation of any substance, whatever, unless it be subjected to certain changes? Vitality, as M. Kopp has remarked, destroys existing combinations, and forms others according to its own laws: this faculty may, it is true, be modified or limited by disease, but it never ceases entirely until after death. We should even regard, as a character of organic action, its power to form compounds more complicated, than those which result from the action of inorganic nature. This action frequently modifies the substances which are received from without, and developes others which the chemists regard as undecomposable. The cones of the pine, growing upon a sterile sand, which does not contain an atom of calcareous earth, have nevertheless yielded that substance to the chemist Einhoff. The observations of M. Lampadius have proved that vegetables contain earths which are not found in the soils where they grow, and the experiments of Braconnet, have left not the least doubt on this point.

Certain assimilated substances preserve, however, some one of their properties, such as their colour, odour, &c. We know, for example, that madder, taken internally, colours the bones red; that logwood communicates to the urine a similar hue; and rhubarb a yellow colour. The mushroom, (*agaric*) with which the inhabitants of Kamtschatca intoxicate themselves, imparts its inebriating quality to the urine. Frictions with garlic give to the breath the odour of that vegetable, &c. But are these substances really in the same state of combination, in which they were, before their ingestion and application; and, in the supposition that alcohol traverses the living body, as it spreads through a sponge, would not its great affinity for water, present an obstacle to its combustion? Besides, do not the inflammable eructations, which have been sometimes observed from brandy drinkers, prove that the liquor had already undergone a change in the stomach, since alcoholic vapours are not susceptible of taking fire, by simple contact with the atmosphere? The dissection of dead bodies is not more conclusive, since brandy, taken in large quantities, a short time before death, might readily transude through tissues, no longer endowed with the resistance of a vital principle. Our supposition is still further supported, by the observations of Metzger, who, in dissecting a great number of drunkards from brandy, of both sexes, never perceived any alcoholic odour, but found the stomach inflamed. Finally, the resemblance of the flame in human combustion, to that of alcohol, cannot be of any weight, since it equally resembles that emitted by several other bodies in a state of combustion, such as hydrogen gas, carbon and sulphur. We may add to these objections, that *every one*, who has perished by spontaneous combustion, has not been addicted to intemperance.

As to the state of corpulence or of leanness, it is certain that the fat in the cellular tissue, is never so destitute of aqueous particles, nor the emaciation so extreme, as to produce a degree of dryness that would favor rapid combustion.

In grouping the various cases of spontaneous human combustion, as M. Lair has done in the work already quoted, for the purpose of deducing from them some general conclusions, we find:

1. That women are a great deal more subject to this accident than men. Now we know that the skin and the cellular tissue of the female sex, are more tender and relaxed, than the male, and that women are more inclined to corpulence.

2. That spontaneous combustion has been confined to aged persons; almost all of them having passed the sixtieth year.

3. That besides the debility of the vital powers incident to this age, the individuals have generally been further reduced by some enervating affection.

4. That they had augmented their delicacy of constitution by an inactive life.

5. That the majority of them have been fleshy, (polysarcous) which, at an advanced age, generally implies atony, especially of the lymphatic system. Dropsy and cachexy are also common among this class of subjects.

6. That the greater part of those who have suffered spontaneous combustion, have been given to the excessive use of ardent spirits.

7. That some burning body, such as a lamp or coal of fire, has, almost always, been found near the place where the accident happened.

8. That the combustion has been so rapid as to extend to the whole body, before assistance could be rendered.

9. That the flame has exhibited great mobility; been difficult to extinguish with water; and has not attacked the surrounding bodies, even when in contact with them for some time.

10. That the spot where the combustion happened, has exhaled a strong empyreumatic odour; that the walls, the the ashes, and the coals, have been covered with a fœtid and greasy moisture.

11. That the trunk of the body has been, almost always, nearly consumed; and that, in the greater number of cases, the fragments remaining have been those of the head and extremities.

12. Finally, that in a great majority of instances, the event has happened in winter, when the atmosphere was cold.

Now, to arrive at an explanation of the phenomenon which occupies us, it is necessary to refer to the combustibility of the body, and to the cause which excites the combustion. The combustibility should, we conceive, be strictly and rigorously considered, since, in the state of health, the human body, is of the class of substances, that are burnt with difficulty.

This combustibility is caused by the weakness of the vital powers, from age, diseases, and a life of inactivity and excesses; the abuse of strong liquors, especially of brandy, debilitates the absorbent system, in a particular manner; and this condition may give origin, in certain cases, to an inflammable substance, which may accumulate in greater or less quantities, in the different parts of the body, according to their varieties of structure.

The combustible substance ought, then, to have the property of easily penetrating the cells of the body, without losing its combustibility by the contact of fluids.

There is not any known body, which more fully unites these conditions, than the inflammable gases; and without their agency, we cannot explain the occurrence to which our attention is directed. For it to take place, such a gas must accumulate in the cells of the body, as lymph accumulates in dropsy; and without insisting on the pre-existence of all the gas necessary to finish the combustion, we may suppose, with great probability, that this operation gives rise to a new gaseous developement, in the parts affected; and that a sufficient quantity is thus generated to finish the combustion. This theory obviates the objection founded upon the absence of a general emphysema in some of the victims of this kind of combustion, a state which appears, occasionally, to have existed.

Hydrogen is a principal element of animal bodies: it manifests itself during life, as well as after death; and forms a great variety of combinations with caloric, carbon, sulphur and phosphorus. Without insisting very much upon this generally admitted truth, it may be useful, at this moment, to recollect the different phenomena, which have a direct and forcible bearing on our subject.

Morton saw a flame project from under the skin of a hog at the moment an incision was made. Bonamet Ruysch, having applied a candle to the vapours which escaped, in an explosive manner, from the stomach of a woman whom he was dissecting, observed them to inflame. Ruysch remarked a similar fact, in examining the stomach of a female, who, for four days before her death, had not taken any nourishment. In other cases the gas has become inflamed without the contact of a burning body, and merely from mixing with the atmosphere. Such, among other examples, is that published in the Memoirs of the Royal Academy of Sciences, of Paris, for 1751. It happened in the same year, in the vicinity of Neufchatel. At the moment when a butcher opened an ox, which had been sick for some time, and was very much swollen, a flame projected from the paunch, which rose to the height of more than five feet. It burnt, not only himself, but a little girl that stood by his side; continued for several minutes, and emitted a very disagreeable odour.

The production, in the system, during life, of hydrogen gas, is not to be doubted. It takes place daily, as we know, in the alimentary canal; and similar observations to those which have been cited, are not rare. Sturm, Nieremberg, Bartholin, Gaubius, and Gmelin, speak of inflammable eructations, which appear to have occurred, principally in the north; where, after the excessive use of brandy, the individuals have been suddenly exposed to a cold atmosphere. The National Gazette of Bohemia announced, a few years since, an accident of this kind, which happened to a shepherd of Lahowitz, who died, in presence of several witnesses, in consequence of an inflammable eructation—the flame resisting every means which could be employed for its extinction. In these different cases, the decomposition of the alcohol and of the animal substances contained in the stomach, gave rise to the phosphuretted hydrogen which inflamed, spontaneously, on coming in contact with the atmosphere. This inflammation was not more extensive, because the other parts of the body were not in a state to favor a general combustion.

Since we cannot deny the developement of inflammable gases, in the living body, we may consistently admit their accumulation in the cellular tissue, to a greater or less extent, according as it is more or less loose in its texture; and of course the parts which are the softest, as the trunk of the body, will be most subject to this accumulation.

But the human body, even rendered thus eminently combustible, cannot be inflamed without the agency of a spark; for, in admitting, that a part of the inflammable principle consists of phosphuretted hydrogen gas, we cannot sufficiently explain what takes place; and, in all cases, the sudden inflammation seems to have been general.

We regard the vicinity of a burning body, as the exciting cause of the combustion; and M. Lair has even concluded that we cannot, strictly speaking, recognise *spontaneous* human combustions, since they are but accidental; but we do not perceive, according to this supposition, how the combustion could be so rapid, so general, and so complete; and we conceive still less at what point it would first show itself. Besides, there are cases of human combustion, where there can be no question of the presence of an ignited body.

In comparing these different considerations with the accident which happened to the priest Bertholi, we are disposed to regard electricity, as performing an important part in human combustions; and must even consider it as the occasional cause of those accidents.

Nobody doubts the idio-electricity of a great number of animals; and this state is also met with in a high degree, in many individuals of the human species. The celebrated traveller Brydone made experiments upon a female, whose idio-electricity was such, that sparks projected from her hair every time she combed it. He was able even to charge a Leyden vial, and to inflame brandy with these sparks. His experiments were made during a period of severe frost. A Senator of the United States, Mr. Jonathan Dayton, when at Washington, remarked, in pulling off and separating at night his woollen and silk stockings, that they emitted electric sparks. But nothing would be easier, than to collect an infinite number of such facts, disseminated through authors, both ancient and modern.

Inflammable substances accumulated in the bodies of the victims of spontaneous combustion, ought, even by their nature, augment the electric state. Overheating the body may contribute, equally, to an explosion of the electric matter; and it is thus that the proximity of fire or of a burning candle, may, in certain cases, aid in human combustion: at other times, this effect may be produced by violent exercise, or by any other cause which can excite electricity.

The electric spark, thus developed, penetrates with extreme celerity a body impregnated with any kind of inflammable matter; which, being every where inflamed, cannot be subdued by the aqueous particles of the system, so that the combustion, in a great number of cases, takes place with such rapidity, that the unfortunate victims have not time to call for assistance. The fire, as observation proves, at first spreads upon the surface, because it is in contact with the atmosphere, and thence extends to the deeper seated parts.

This theory will explain, without much difficulty, the following circumstances:

Women are more subject than men to spontaneous combustion, because their tissues are less compact, and, consequently, more disposed to gaseous accumulations.

Spontaneous combustions occur most frequently among the aged, because they are more addicted than the young, to the use of spirituous liquors; they take but little exercise, and their vital energy, especially in the lymphatic system, is enfeebled.

The character of the flame—its lightness, its mobility, and its resistance to the action of water—are all proper to hydrogen gas. In some phenomena of nature, such as the ignis fatuus, and certain meteors, in which this gas is supposed to perform an important part, it manifests the same properties.

The furniture and other surrounding objects are but little injured, because hydrogen gas, in combustion, does not inflame the greater number of bodies, unless it be in very close contact with them.

The water which bedews the walls of the apartment in which the combustion took place, as well as the coals and ashes of the deceased, is furnished by the combustion of hydrogen gas; and, also, by the evaporation of the liquids of the burnt body. The unctuous aspect of this water, is owing to the presence of a portion of fat, which has been volatilized by the heat.

The fœtid smell is that of the empyreumatic oil, formed during the combustion.

The trunk of the body is always more injured than the other parts, because of the great size of its cavities; and of the looseness of their cellular texture. Finally—

The winter is the season when spontaneous human combustions most frequently occur; because the cold air is a bad conductor of electricity, and promotes the idio-electricity of the animal body.

—

### EDITORIAL NOTE.

We have translated (hastily and inelegantly) the ingenious memoir of M. Marc, as a kind of supplement to the Discourse on Intemperance, published in the preceding numbers of this Journal. Although a translation and reprint, it will, we presume, offer considerable novelty to most of our readers, as the subject is one which has attracted but little attention from British and American writers. Indeed, of nine works referred to by M. Marc at the end of his article, every one is Continental. Whether this should be regarded as evidence that this event is more frequent among the people of the Continent, than those of Great Britain and the United States, or only that it has received more attention, we are unable to say.

